# Propofol Suppressed Hypoxia/Reoxygenation-Induced Apoptosis in HBVSMC by Regulation of the Expression of Bcl-2, Bax, Caspase3, Kir6.1, and p-JNK

**DOI:** 10.1155/2016/1518738

**Published:** 2016-01-05

**Authors:** Jianhai Zhang, Yunfei Xia, Zifeng Xu, Xiaoming Deng

**Affiliations:** ^1^Department of Anesthesiology, Shanghai First People's Hospital, Shanghai Jiao Tong University, Shanghai 200080, China; ^2^Department of Anesthesiology, Shanghai International Medical Center, Shanghai 201318, China; ^3^Department of Anesthesiology, International Peace Maternal and Child Health Hospital, Shanghai Jiao Tong University, Shanghai 200030, China; ^4^Department of Anesthesiology, Changhai Hospital, Second Military Medical University, 168 Changhai Road, Shanghai 200433, China

## Abstract

Recent studies have found that propofol may protect brain from cerebral ischemic-reperfusion injury. However, the underlying mechanism remains unclear. The effects of propofol were evaluated in HBVSMC after hypoxia/reoxygenation (H/R). Cell viability and levels of SOD, LDH, and MDA were measured. Apoptosis was detected by flow cytometry. The levels of Bax, Bcl-2, Caspase3, Sur2b, Kir6.1, JNK, p-JNK, mTOR, and p-mTOR proteins were measured by western blotting. H/R decreased cell viability and SOD activity and increased LDH leakage and MDA content in HBVSMC, all of which were significantly reversed by propofol. Propofol suppressed the levels of H/R-induced apoptosis. The expression of Bcl-2 and p-mTOR was significantly downregulated and the expression levels of Bax, Caspase3, Kir6.1, and p-JNK were upregulated following H/R injury. The ratio of p-JNK/JNK was increased; however, that of p-mTOR/mTOR decreased correspondingly. The effects on the expression of these proteins were reversed by propofol treatment. SP600125 enhanced and Everolimus attenuated the effect of propofol. These findings suggested that the protective effect of propofol against H/R injury in the HBVSMC was through the inhibition of apoptosis by inducing the expression of Bcl-2 and p-mTOR as well as inhibiting the expression levels of Bax, Caspase3, Kir6.1, and p-JNK.

## 1. Introduction

Transient global cerebral ischemia is one of the major complications of clinical emergencies such as cardiac arrest, drowning, or severe systemic hypotension during a surgical procedure [1]. Ischemic hypoxic brain injury often causes irreversible brain damage. Ischemic stroke accounts for approximately 80% of all strokes [2] which remains a leading cause of death and adult disability worldwide [3]. Currently, reperfusion of the occluded vessels as soon as possible is the standard treatment for these patients. However, reperfusion may paradoxically exacerbate brain injury, which is called cerebral ischemia/reperfusion (I/R) injury [4]. Ischemic stroke is a serious human health risk and reperfusion plays an important role in cerebral ischemic injury. The extent of brain damage is determined by the severity of primary injury and the intensity of secondary injury cascades that contribute to delayed cellular destruction [5]. Ischemic-reperfusion injury leading to neuronal injury and death includes the release of cytokines and free radicals and induction of inflammation, apoptosis, and excitotoxicity [6]. Apoptosis and oxidative stress have been found to play an important role in the pathogenesis of cerebral injury secondary to ischemia/reperfusion (I/R) [7, 8]. The striking relationship between apoptosis and brain I/R injury has stimulated considerable interest in the development of antiapoptosis therapies [9, 10]. Therefore, efforts need to be made that not only preserve cerebral blood flow, but also prevent the actual mechanisms that trigger brain damage after I/R injury [11].

Propofol (2,6-disopropylphenol) is an intravenous sedative-hypnotic agent. It is widely used in clinical anesthesia and maintenance of anesthesia or sedation. Recent studies have found that propofol as one of the central inhibitors could reduce brain oxygen consumption and increase intracranial pressure (ICP) and has anticonvulsant, anti-inflammatory, and antioxidant activities. It could also relieve the neurosurgery postoperative damage of brain tissue and blood vessel. However, the mechanism of propofol's protective effect on cerebral hypoxia is not very clear. The object of our study is to explore the mechanism of propofol against cerebral ischemic-reperfusion injury* in vitro*.

## 2. Materials and Methods

### 2.1. Cell Culture

Human brain vascular smooth muscle cells (HBVSMC) were purchased from the Cell Bank of Chinese Academy of Sciences (Shanghai, China). The cells were cultured in Dulbecco's modified Eagle medium (DMEM) supplemented with 10% fetal bovine serum both from GIBCO-Invitrogen (Grand Island, NY) and 1% penicillin and streptomycin. Cells were maintained at 37°C in a humidified atmosphere consisting of 5% CO_2_ and 95% air.

### 2.2. Hypoxia/Reoxygenation (H/R) Model and Drug Treatment

Cells were exposed to hypoxic conditions (oxygen deprivation, 0.5% O_2_) for 2 h, 4 h, 6 h, and 8 h in culture. After hypoxia, the cells were reoxygenated under normoxic conditions (reoxygenation) for 16 h in normal medium [12]. Propofol (Fresenius Kabi, China) with different concentrations (25, 50, and 100 *μ*M) or propofol combined with SP600125 (Sigma-Aldrich, St. Louis, MO, USA)/Everolimus (Gene Operation Michigan, USA) was added to the cells prior to hypoxia.

### 2.3. Cell Viability Assay

Cells were plated in 96-well plates at 1 × 10^4^ cells/well. After 24 h of culture, cells were under hypoxia-oxygenation for several hours or treated with different concentrations of propofol followed by hypoxia-oxygenation or treated with propofol or propofol combined with SP600125/Everolimus for hypoxia-oxygenation and then cultured for 1 d, 2 d, 3 d, 4 d, 5 d, and 6 d in normal conditions, respectively. Cell viability was assessed using Cell Counting Kit-8 (Beyotime, Shanghai, China). 20 *μ*L of cell counting assay kit-8 solution was added daily to three wells per group. After treatment with CCK-8 at 37°C for 2 h, the absorbance at 450 nm was measured using a microplate reader to quantify the formazan products.

### 2.4. Measurement of SOD, LDH, and MDA by ELISA

Superoxide dismutase (SOD) activity was assayed with an assay kit purchased from Jiancheng Co. (Nanjing, China). After being frozen and thawed using liquid nitrogen repeatedly, cells were moved to EP tube with hypotonic solution or distilled water. The tube was directly put into liquid nitrogen for 3–5 seconds, followed by −20°C refrigerator (20–30 seconds) immediately, and then thawed at room temperature. The previous process was repeated three times. SOD activity was measured according to the kit's instructions of the manufacturer.

The level of lactate dehydrogenase (LDH) was measured using LDH Activity Assay Kit (Jiancheng Co., Nanjing, China). Culture medium was collected and transferred to a 6-well plate. LDH reaction mix was added to each well, and the plates were incubated for 30 min at 37°C and for 5 min at room temperature. The absorbance was read at 450 nm when the reaction was stopped.

MDA assay was determined by lipid peroxidation MDA Assay Kit (Beyotime, Shanghai, China). Cells were lysed and reacted with thiobarbituric acid (TBA). The product has an absorbance peak at 532 nm. MDA was calculated by using a standard curve according to the manufacturer's data sheet. The results were expressed as micromole per gram protein (*μ*mol/g protein).

### 2.5. Apoptosis Assay by Flow Cytometry

Cells after H/R and treatment with propofol (50 *μ*M) or SP600125/Everolimus were harvested by brief trypsinization and centrifugation (at 170 ×g), washed in ice-cold PBS, and fixed in 70% ethanol for 2 hours at −20°C. Apoptosis was detected by double-staining with annexin V conjugated to fluorescein isothiocyanate and propidium iodide (Bender Medsystems). Ten thousand cells per sample were acquired in a FACScan flow cytometer and the proportions of labeled cells were analyzed using Paint-A-Gate software (Becton Dickinson).

### 2.6. Western Blot Analysis

Cells after H/R and treatment with propofol or SP600125/Everolimus were harvested and washed twice with cold PBS and subsequently lysed in 1x SDS-PAGE loading buffer (120 *μ*L per well of 6-well plate). The samples of cell lysis were heated to 95–100°C for 10 min followed by cooling on ice and centrifuged at 10,960 ×g for 1 min at 4°C. The supernatant was run on 10% SDS-PAGE gel and transferred electrophoretically to a polyvinylidene fluoride membrane (PVDF, Millipore, Shanghai, China). The blots were blocked for 1 h at 25°C with 5% skim milk in Tris-buffered saline containing Tween 20 (TBST) followed by incubation with primary antibodies against Tubulin (Abcam), Bax (Proteintech), Bcl-2 (Abcam), Caspase3 (CST), Sur2b (CST), Kir6.1 (Santa Cruz Biotechnology), JNK (CST), p-JNK (CST), mTOR (CST), and p-mTOR (CST) overnight at 4°C. After being washed with TBST, the membranes were incubated with proper secondary antibodies (Beyotime, Shanghai, China). Blots were then incubated and visualized with enhanced chemiluminescence (ECL, Thermo Scientific, Shanghai, China). The results were normalized to Tubulin to correct for loading.

### 2.7. Data Analysis

Results are presented as means ± standard deviations (SD) of three samples. Significant differences in the mean values were evaluated by Student's unpaired* t*-test. Places needing multiple comparisons were evaluated by one-way ANOVA with Bonferroni correction. *P* value of 0.05 or less was considered to be statistically significant.

## 3. Results

### 3.1. Propofol Inhibited Cell Viability Decrease

**Figure 1:**
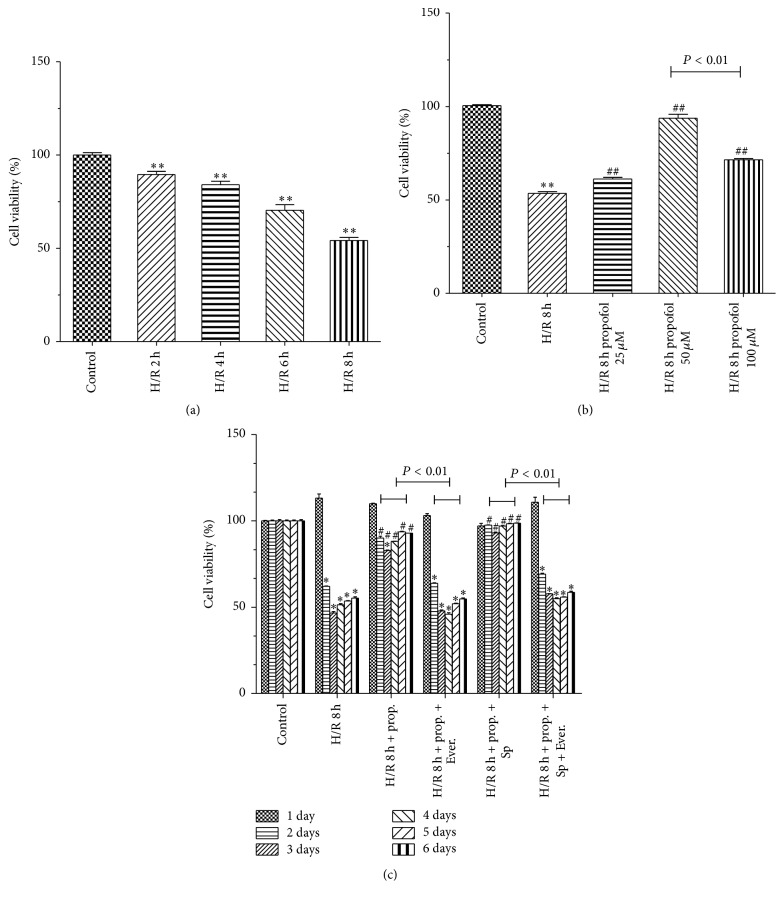
Protective effects of propofol on H/R-induced cytotoxicity HBVSMC. (a) Cell viability was assessed by CCK8 assay. Cells were exposed to hypoxic conditions. Hypoxia/reoxygenation (H/R) for 2 h, 4 h, 6 h, and 8 h. The viability of control group was defined as 100%. (b) Propofol (25, 50, and 100 *μ*M) was adopted during the entire ischemia/reperfusion phase. Cell viability was assessed. (c) HBVSMC after H/R and treatment with propofol or propofol combined with SP600125 (SP)/Everolimus (Ever.) were cultured for 1 d, 2 d, 3 d, 4 d, 5 d, and 6 d in normal conditions. Cell viability was assessed. Mean ± SD, *n* = 3. ^*∗*^ *P* < 0.05 versus control. ^#^ *P* < 0.05 versus H/R group without drugs.

Compared with control group, cell viability was significantly decreased during H/R insult in model group (Figure 1(a)). Compared with model group, propofol treatments significantly inhibited the decrease of cell viability (Figure 1(b)). Propofol inhibited the cell damage in a dose-dependent manner induced by H/R. However, 100 *μ*M of propofol has damaging effects on HBVSMC under oxidative stress conditions.  Figure 1(c) shows the viability of HBVSMC after addition of propofol combined with SP600125/Everolimus to the cells prior to hypoxia. Everolimus, a mTOR inhibitor, blocked the effect of propofol on the cells viability induced by H/R. Cell viability of propofol-SP600125 group was higher than that of propofol group (*P* < 0.01). The effect of SP600125, a JNK inhibitor, as reported [13] was significantly attenuated by Everolimus.

### 3.2. Propofol Decreased LDH and MDA Levels Induced by H/R in HBVSMC

**Figure 2:**
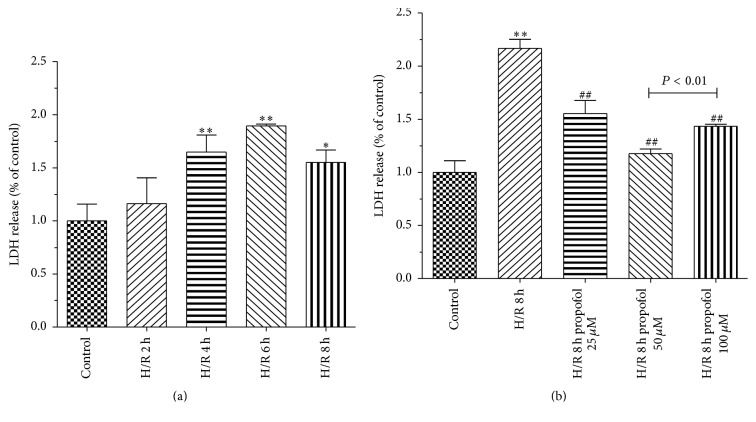
Effects of propofol on LDH leakage in HBVSMC. (a) Cells were exposed to hypoxic conditions. Hypoxia/reoxygenation (H/R) for 2 h, 4 h, 6 h, and 8 h. (b) Propofol (25, 50, and 100 *μ*M) was adopted during the entire ischemia/reperfusion phase. Mean ± SD, *n* = 3. ^*∗*^ *P* < 0.05, ^*∗∗*^ *P* < 0.01 versus control; ^#^ *P* < 0.05, ^##^ *P* < 0.01 versus H/R group without drugs.

H/R is known to induce oxidative stress. Cell death was assessed based on the amount of lactate dehydrogenase (LDH). Compared with model group, propofol treatments significantly inhibited LDH leakage induced by H/R (Figures 2(a) and 2(b)).

**Figure 3:**
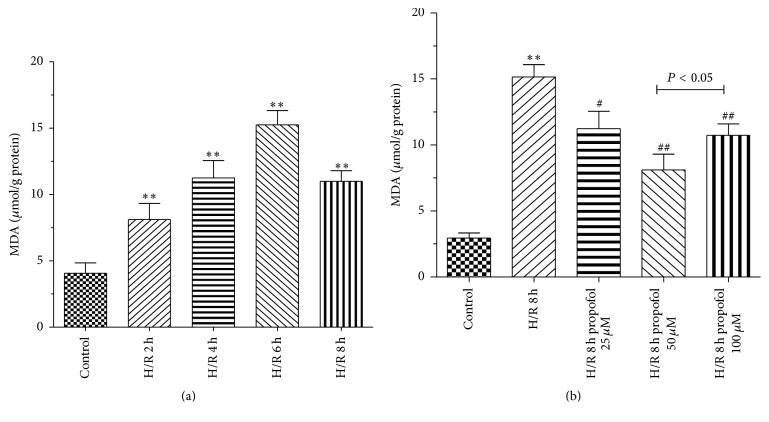
Effects of propofol on MDA in HBVSMC. (a) Cells were exposed to hypoxic conditions. Hypoxia/reoxygenation (H/R) for 2 h, 4 h, 6 h, and 8 h. (b) Propofol (25, 50, and 100 *μ*M) was adopted during the entire ischemia/reperfusion phase. Mean ± SD, *n* = 3. ^*∗*^ *P* < 0.05, ^*∗∗*^ *P* < 0.01 versus control; ^#^ *P* < 0.05, ^##^ *P* < 0.01 versus H/R group without drugs.

MDA, which is a marker of lipid peroxidation, was measured to evaluate the oxidative injury of H/R. In our study, H/R obviously elevated intracellular MDA levels compared with control. Compared with H/R group, propofol obviously inhibited MDA levels (Figures 3(a) and 3(b)). Moreover, the levels of MDA were measured as an indicator of lipid peroxidation. The results showed that propofol (25, 50, and 100 *μ*M) could inhibit lipid peroxidation injury in cells. The result was consistent with previous report about propofol decreasing the levels of MDA [14, 15].

### 3.3. Propofol Increased the Activities of SOD in H/R Group

**Figure 4:**
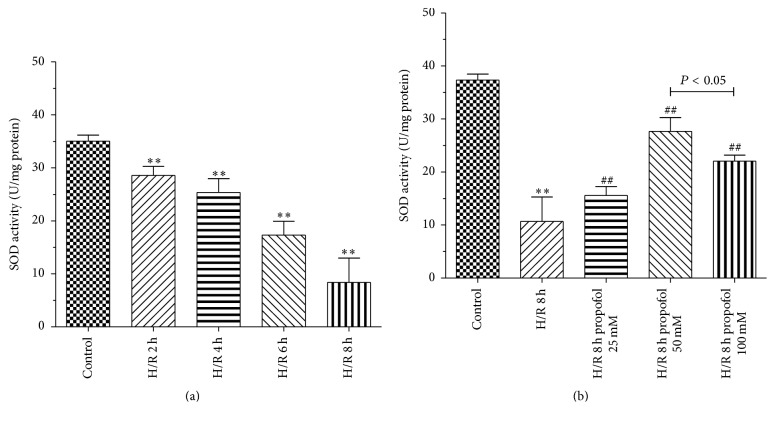
Effects of propofol on SOD activity in HBVSMC. (a) Cells were exposed to hypoxic conditions. Hypoxia/reoxygenation (H/R) for 2 h, 4 h, 6 h, and 8 h. (b) Propofol (25, 50, and 100 *μ*M) was adopted during the entire ischemia/reperfusion phase. Mean ± SD, *n* = 3. ^*∗*^ *P* < 0.05, ^*∗∗*^ *P* < 0.01 versus control; ^#^ *P* < 0.05, ^##^ *P* < 0.01 versus H/R group without drugs.

Superoxide dismutase (SOD) can protect cells from damage by elimination of oxygen free radicals. SOD is one of the endogenous antioxidative enzymes that protect against ROS-induced damage [15]. In H/R model group, the activities of SOD were significantly decreased compared with control group. Propofol (25, 50, and 100 *μ*M) group significantly increased SOD activity (Figures 4(a) and 4(b)).

### 3.4. HBVSMC Apoptosis

**Figure 5:**
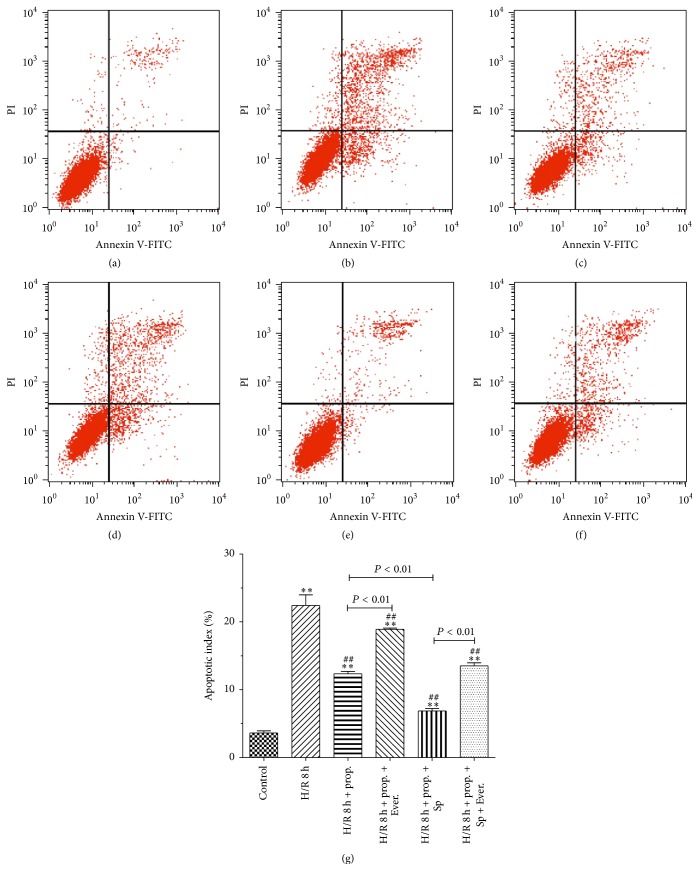
Effects of propofol on apoptosis of HBVSMC. (a)–(f) Representative figures of flow cytometry results and rate of apoptosis measured by flow cytometry (g). Flow cytometric analysis was carried out as described in Materials and Methods. Primary cultured HBVSMC were either not treated (control) or treated with propofol (50 *μ*M) or propofol (50 *μ*M) combined with SP600125 (SP)/Everolimus (Ever.). Mean ± SD, *n* = 3. ^*∗*^ *P* < 0.05, ^*∗∗*^ *P* < 0.01 versus control; ^#^ *P* < 0.05, ^##^ *P* < 0.01 versus H/R group without drugs.

Stimulation of HBVSMC by H/R resulted in a marked significant increase in apoptotic index (Figure 5). Propofol decreased the rate of apoptotic cells. SP600125 decreased H/R-induced apoptotic cell death. On the other hand, Everolimus significantly increased cell apoptosis induced by H/R. Propofol combined with SP600125 further attenuated cell apoptosis induced by H/R. Propofol attenuated but did not prevent apoptotic cell death induced by Everolimus in combination with H/R.

### 3.5. Protein Expression of Bax, Bcl-2, Caspase3, Sur2b, Kir6.1, JNK, p-JNK, mTOR, and p-mTOR

**Figure 6:**
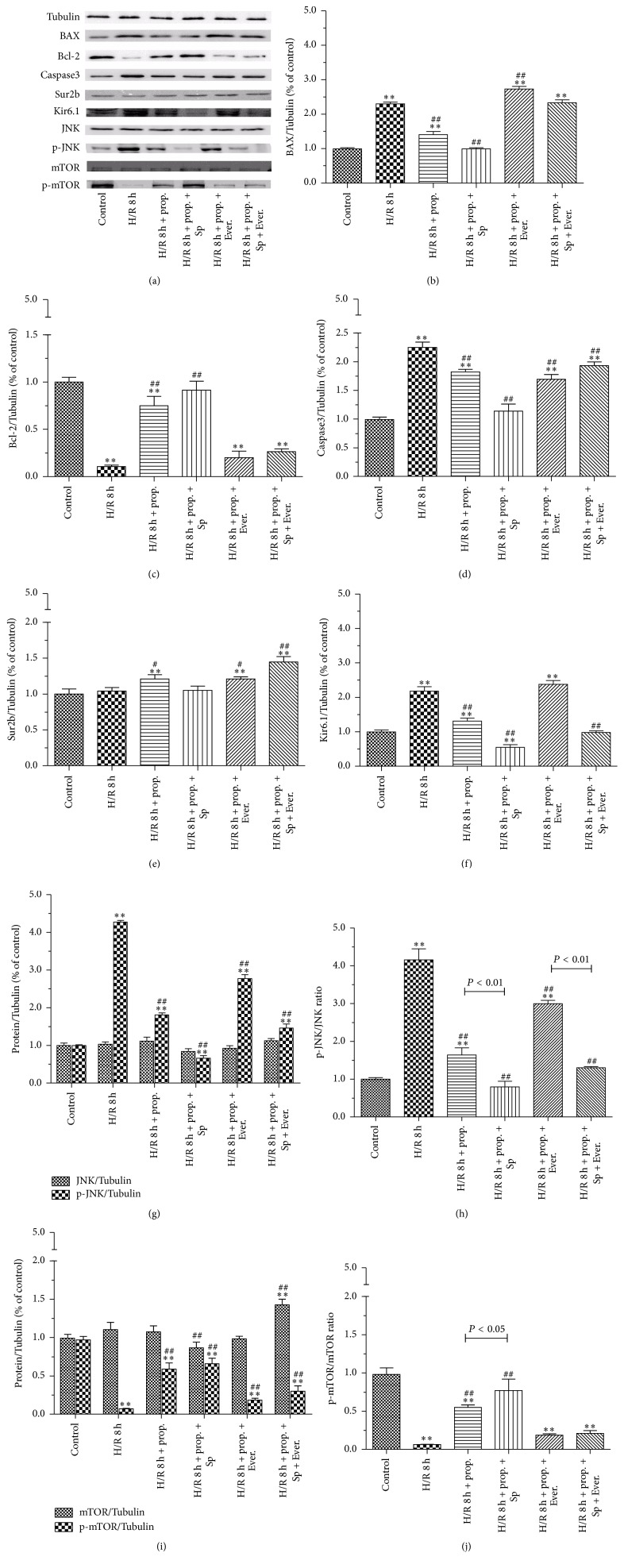
The effects of propofol (50 *μ*M) on Bax, Bcl-2, Caspase3, Sur2b, Kir6.1, JNK, p-JNK, mTOR, and p-mTOR expression in HBVSMC. (a)–(f), (g), and (i) represent the levels of Bax, Bcl-2, Caspase3, Sur2b, Kir6.1, JNK, p-JNK, mTOR, and p-mTOR which were determined by western blotting and Tubulin was used as positive control. (h) and (j) show the ratio of p-JNK/JNK and p-mTOR/mTOR, respectively. HBVSMC cells were either not treated (control) or treated with propofol (50 *μ*M) or propofol (50 *μ*M) combined with SP600125 (Sp)/Everolimus (Ever.). Mean ± SD, *n* = 4. ^*∗*^ *P* < 0.05, ^*∗∗*^ *P* < 0.01 versus control; ^#^ *P* < 0.05, ^##^ *P* < 0.01 versus H/R group without drugs.

To investigate the effect of propofol on the expression of HBVSMC hypoxia/reoxygenation injury-related proteins and the mechanism involved, we measured the levels of Bax, Bcl-2, Caspase3, Sur2b, Kir6.1, JNK, p-JNK, mTOR, and p-mTOR proteins. As shown in Figure 6, the protein expressions of Bax, Caspase3, Kir6.1, and p-JNK in H/R group were 1.30, 1.25, 1.18, and 3.27 times more than that in control group, which were attenuated by the treatment of propofol. The protein expressions of p-JNK and the ratio of p-JNK/JNK were especially high. Addition of Everolimus further decreased the protein expression of Bax, Caspase3, Kir6.1, and p-JNK, which was reversed by SP600125 treatment. The protein expressions of Bcl-2 and p-mTOR were obviously decreased while the ratio of p-mTOR/mTOR was decreased correspondingly in HBVSMC subjected to H/R stimulation as compared with those of control group, which were attenuated by the treatment of propofol. Propofol combined with Everolimus further increased the protein expression of Bcl-2 and p-mTOR, which was reversed by SP600125 treatment. Furthermore, there was no significant difference in the protein expressions of Sur2b, JNK, and mTOR between H/R and control group.

## 4. Discussion

In the present study, we used human brain vascular smooth muscle cell (HBVSMC) to establish H/R model* in vitro* to investigate the effect of propofol in the treatment of ischemia/reperfusion (I/R) injury. Vascular smooth muscle cell is the cellular substrate of most significant arterial diseases [16]. One of the crucial anomalies responsible for the development of essential vascular diseases is the increased growth potential of vascular smooth muscle cells. Smooth muscle cells proliferation in atherosclerosis was of particular concern as a high proportion of cardiac and central nervous system death is believed to result from spasm of damaged vessels [16]. Cerebral vasculature plays a central role in the pathogenesis of cardiovascular diseases, such as stroke [17, 18], and in the initiation of inflammation after cerebral ischemia, which is a key determinant in stroke outcome [18, 19]. A substantial source of reactive oxygen species in the site of ischemic lesion is also the immune response [17]. Vascular smooth muscle cell, smooth muscle actin (SMA), was one of the blood brain barrier components [20, 21]. Experimental results have shown that ischemia followed by reperfusion results in blood brain barrier disruption [22–24] and leakage of immune cells into the damaged brain tissue [25–27]. I/R injury is the tissue injury, structure damage, and dysfunction of organs due to the reperfusion after ischemia. As a secondary injury after primary brain injury, I/R injury is an important factor that contributes to the brain injury. Besides the significant decrease of brain function, it can cause secondary injury of heart, liver, and kidney, being seriously harmful to the whole body [28–30]. The incidence rate of I/R injury has also increased as the natural disasters and traffic accidents increase in recent years [31]. With the rapid development of medical technology, there have been several methods to decrease the risk of I/R injury, such as reducing the operation time, improving the operation method, preadaptation to ischemia, and increasing blood supply for brain; however, there is no obvious change of biochemical factors inducing I/R injury [32]. In recent years, numerous studies found that the excellent effect of propofol on anti-inflammation and antioxidation could inhibit the overrelease of oxygen radical under stress status, which was expected to reduce the risk of biochemical factor mediated I/R injury [33–35]. And several studies have found that propofol had effects on the prevention and treatment of I/R injury; however, further exploration about the mechanism is still needed [36, 37]. As previously reported [38, 39], the change of protein physicochemical property, structure, and function is the important reason which causes occurrence and development of I/R injury. Therefore, we explored I/R injury-related protein expression and mechanism expected to decrease the incidence rate of I/R injury.

We examined protective effects and mechanism of propofol against H/R-induced injury in HBVSMC in the presence or absence of SP600125/Everolimus. Hypoxia/reoxygenation injury was assessed by using the cell viability, SOD activity, LDH leakage, and MDA content. Results showed that cell viability and SOD activity decreased with prolonged H/R injury. LDH leakage and MDA content were the highest at 6 h. The most possible reason was that enhanced resistance to H/R and cells self-recovery from injury increased at 8 h [40]. In order to explore the effect of propofol against H/R injury, we selected 8 h after hypoxia for further research. In our study, the data demonstrated that treatment with propofol reduced cell death, LDH leakage, and MDA content and increased SOD activity in a dose-dependent way, which indicate that propofol has a strong protective effect against oxidative stress induced injury in HBVSMC. However, cell death, LDH leakage, and MDA content increased and SOD activity decreased when the concentration of propofol was 100 *μ*M, which suggested that 100 *μ*M propofol exhibited cytotoxicity* in vitro* in HBVSMC. Propofol is highly lipophilic and therefore is concentrated in lipid-rich tissues such as brain [41, 42]. Studies in animals [42] and humans [41, 43, 44] indicate that the measured/predicted brain concentration of propofol during maintenance of surgical anesthesia is above 22 *μ*M and as high as 73 *μ*M. The 25–50 *μ*M concentration range used in our experiments is therefore within the range of concentrations that exist in the human brain during anesthesia; however, 100 *μ*M is higher than the upper limit of the concentration range. As previously reported [45], overdose of propofol (140 *μ*M) causes GSK-3*β*-mediated macrophage apoptosis; however, the attenuated effect of propofol with low dose (5.6–56 *μ*M) has been shown. Liu et al. [46] reported that 100 *μ*M of propofol and/or higher doses (e.g., 300 and 600 *μ*M) decreased rat neural stem cell viability. Another previous report also showed that 50 *μ*M of propofol has damaging effects on H9c2 cells under oxidative stress conditions [47]. The damaging effects of propofol (100 *µ*M) on HBVSMC cells under oxidative stress conditions may be similar to propofol effects on macrophage, rat neural stem cell, and H9c2 cells. Therefore, we used 50 *μ*M propofol for further study. Everolimus, as a mTOR inhibitor, blocked the effect of propofol on the cells viability induced by H/R significantly compared with JNK inhibitor SP600125. Propofol combined with SP600125 further decreased the apoptosis of HBVSMC which suggested that SP600125 enhanced the protective effect of propofol against H/R injury. Propofol combined with Everolimus further increased the apoptosis of HBVSMC and the index of the apoptosis of HBVSMC in group treated with propofol combined with SP600125 and Everolimus was between propofol combined with SP600125 and propofol combined with Everolimus. Decreased cell viability was a result of increased cell apoptosis. In our study, the result of cell apoptosis was consistent with that of cell viability. According to previous reports, activation of the JNK signaling cascade and expression of Bcl-2, Bax, and Caspase3 leads to apoptosis during H/R [40, 48]; mTOR function was one of the “master switch” proteins in cells to modulate metabolism, cell cycle, and apoptosis [49]; Sur and Kir subunits are able to associate to form potassium channels [50, 51], which associate with apoptosis. We measured the levels of Bax, Bcl-2, Caspase3, Sur2b, Kir6.1, JNK, p-JNK, mTOR, and p-mTOR proteins to investigate the mechanism of propofol. Results of our study suggested that the protective effect of propofol against H/R injury in the HBVSMC was through the inhibition of apoptosis by inhibiting the expression levels of Bax, Caspase3, Kir6.1, and p-JNK and inducing the expression of Bcl-2 and p-mTOR.

## 5. Conclusion

In summary, the results from the present study indicated that the protective effect of propofol against H/R injury in the HBVSMC was through the inhibition of apoptosis by inhibiting the expression levels of Bax, Caspase3, Kir6.1, and p-JNK and inducing the expression of Bcl-2 and p-mTOR. Propofol may offer a promising therapeutic approach for the treatment of HBVSMC injury resulting from ischemia/reperfusion (I/R).
